# How to minimise the effect of tumour cell content in detection of aberrant genetic markers in neuroblastoma

**DOI:** 10.1038/bjc.2011.188

**Published:** 2011-06-07

**Authors:** M Piqueras, S Navarro, A Cañete, V Castel, R Noguera

**Affiliations:** 1 Department of Pathology, Medical School, University of Valencia, Avda. Blasco Ibáñez, 17, 46010 Valencia, Spain; 2 Paediatric Oncology Unit, Hospital Infantil Universitario La Fe, Avda. Campanar, 21, 46009 Valencia, Spain

**Keywords:** neuroblastoma, aberrant genetic markers, FISH, tumour cell content, prognostic factors

## Abstract

**Background:**

Clinical heterogeneity reflects the complexity of genetic events associated with neuroblastoma (NB). To identify the status of all described genetic loci with possible prognostic interest, high-throughput approaches have been used, but only with tumour cell content >60%. In some tumours, necrotic, haemorrhagic and/or calcification areas influence the low amount of neuroblasts. We evaluated the effect of tumour cell content in the detection of relevant aberrant genetic markers (AGM) diagnosed by fluorescence *in situ* hybridisation (FISH) on tissue microarrays (TMA) in NB.

**Methods:**

Two hundred and thirty-three *MYCN* non-amplified primary NB included in 12 TMAs were analysed.

**Results:**

Presence of AGM reduced event-free survival (EFS) (*P*=0.004) as well as overall survival (OS) (*P*=0.004) of patients in the whole cohort. There were no differences in prognostic impact of presence of AGM according to tumour cell content.

**Conclusion:**

We propose the use of FISH to diagnose AGM of all NB samples having the above-mentioned areas to determine patient risk.

Neuroblastoma (NB) is the most common cancer diagnosed during the first year of life (Maris, 2010), and accounts for 7.6% of all childhood malignancies (Kaatsch, 2010). This embryonal tumour of the autonomic nervous system is the most frequent extracranial solid cancer; and the clinical presentation is highly variable, ranging from mass without symptoms to widely disseminated disease (Maris, 2010).

Clinical markers are not sufficient for an accurate prediction of prognosis, and genetic markers have been shown to provide prognostic information. The extreme clinical heterogeneity reflects the complexity of genetic and genomic events associated with the development and progression of NB (Capasso and Diskin, 2010). In addition to the evaluation of known single molecular markers such as *MYCN* amplification and 11q deletion, high-throughput approaches have been used. Janoueix-Lerosey *et al* (2009) revealed two distinct genetic classes of NB. Tumours with only numerical chromosome alterations (NCA) are associated with an excellent outcome, even in patients older than 18 months or with advanced stages of disease. The second group includes segmental chromosome alterations (SCA) alone or with *MYCN* amplification. These alterations also occur in association with NCA, but the presence of SCA overrides NCA with respect to prognostic impact. The authors suggested that any SCA is associated with an increased risk of relapse (Janoueix-Lerosey *et al*, 2009). Also, it has been suggested that tumour progression is linked to the accumulation of SCA. This possible genomic evolution should be taken into account in treatment therapies of low- and intermediate-risk NB (Schleiermacher *et al*, 2010).

In this same journal, the International Neuroblastoma Risk Group (INRG) Biology Subcommittee recommends fluorescence *in situ* hybridisation (FISH) to establish the *MYCN* status, whereas segmental aberrations are currently detected using either FISH or polymerase chain reaction (Ambros *et al*, 2009). Since the pattern of DNA-based genomic changes seems to be prognostic, the INRG Biology Subcommittee suggests using pan- or multigenomic techniques, such as array-based methods or multiplex ligation-dependent probe amplification (MLPA), enabling an analysis of all relevant genomic loci (Ambros *et al*, 2009).

However, solid tumour samples show intratumoural heterogeneity, containing tumour cells, as well as normal surrounding and infiltrating somatic tissues, thus complicating the detection of genetic abnormalities (Attiyeh *et al*, 2009). Although necrotic, haemorrhagic and/or calcification areas can reduce the quality of DNA, stromal contamination is the major confounding factor in the analysis of solid tumour samples by DNA-based techniques (Volchenboum *et al*, 2009). Therefore, molecular studies on neuroblastic tumours require identification of tumour and Schwannian stromal cells. The tumour cell content must be determined in collaboration with the pathologist because a content of >60% is an indispensable requisite for most molecular studies using DNA-based methods (Ambros *et al*, 2009, 2011).

Nevertheless, there are samples from neuroblastic tumours containing <60% of neuroblasts. In these cases, a low tumour cell number can also be sufficient to determine aberrant (numeric or structural) genetic markers (AGM) by FISH (Ambros *et al*, 2009). As a combined molecular and cytological approach, the major advantage of this technique is to provide an intermediate degree of resolution between DNA analysis and chromosomal investigations while retaining information at single-cell level (Volpi and Bridger, 2008). The combination of DNA-specific probes for FISH and tissue microarrays (TMAs) facilitates the identification of changes at any genomic locus in several tissue samples at once, independently of the percentage of tumour cells (Piqueras *et al*, 2009, 2010; Sugimura *et al*, 2010).

The aim of this study is to evaluate the effect of tumour cell content on detection of AGM diagnosed by FISH on TMA in NB.

## Materials and methods

Two hundred and thirty-three *MYCN* non-amplified primary NB included in 12 previously constructed TMAs were analysed (Piqueras *et al*, 2010) (Supplementary Table 1, summary of clinical characteristics of patients, supplementary materials available online). Representative tumour areas on haematoxylin and eosin-stained slides, avoiding necrotic, haemorrhagic and artefactual areas, as well as stromal areas with predominantly Schwann cells, were selected by the pathologist. Informed consent was provided by a parent or guardian for each patient enrolled in this study. *MYCN* gene and 1p36 region status, as well as aberrations in 11q arm and 17q arm, had previously been determined by FISH using the following commercial DNA probes: *MYCN* (2p24) red/*α*-satellite 2 green; 1p36 midisatellite green/chromosome 1 satellite red (Q-BIOgene, Amsterdam, The Netherlands); *ATM* (11q22) red/SE 11 green; and *MPO* (17q23) iso 17q red/*p53* (17p13) green (Kreatech Biotechnology, Amsterdam, The Netherlands).

Following our own system for detecting genetic alterations in formalin-fixed paraffin-embedded by FISH, we distinguished cells without any gene alterations (group 1), cells with any possible genetic alteration (group 2) and cells with cutting artefacts (group 3), including nuclear fragments generated from sectioning of the nuclei (Piqueras *et al*, 2009). As previously published, genetic alteration was diagnosed when the net percentage of cells with genetic alteration, obtained subtracting the total percentage of group 3 from the cellular population belonging to group 2, was >15%, (Piqueras *et al*, 2009). ENQUA and INRG definitions for chromosome aberrations in neuroblastic tumours were applied (Ambros *et al*, 2003, 2009).

We considered the status of the four genetic markers. A new variable was created: presence of AGM, distinguishing between tumours with no AGM *vs* tumours with any AGM detected by FISH on TMAs.

### Statistical analysis

Events for event-free survival (EFS) analysis were considered as relapse or death from disease. Time to event for EFS was calculated as the time from diagnosis until the time of first event, or until the time of last patient contact if no event occurred. Time to event for overall survival (OS) analysis was the time from diagnosis until death, or until the time of last contact if the patient was alive. Univariate analyses were performed using Kaplan–Meier to generate survival curves, which were compared using a log-rank test to identify statistically significant predictive factors of EFS and OS. *P*-values <0.05 were considered statistically significant. All EFS and OS values are reported at the 5-year time point with the standard error.

## Results

One third of the 233 patients presented advanced stage disease, and 68% of patients were younger than 18 months. The majority of cases were poorly differentiated or undifferentiated NBs. Regarding genetic alterations diagnosed by FISH on TMAs, 5.6% were *MYCN* gain tumours, 13.3% presented 1p36 deletion, 14.2% harboured 11q deletion and 34.9% showed 17q gain. Ninety-four samples (40.3%) were classified as tumours with AGM; 59 of these had only one genetic alteration, 24 cases had 2 aberrations and 11 had more than 2 AGM.

Presence of AGM reduced EFS (*P*=0.004) as well as OS (*P*=0.004) of patients in the whole cohort. We considered the tumour cell content of the samples and divided the cohort into two subgroups: tumours up to 50% (26.2%) and tumours with >50% of tumour cells (73.8%). Both subgroups included NBs with different degrees of differentiation, as well as ganglioneuroblastomas and ganglioneuromas (Supplementary Table 1, supplementary materials). In the first group, patients harbouring tumours with AGM had significantly worse 5-year EFS than patients with no AGM (64.2±11.9% *vs* 89.5±5.8%, log-rank, *P*=0.028). Also, a trend towards lower 5-year OS (75.6±10.7% *vs* 92.2±5.4%, log-rank, *P*=0.065) was observed. Similarly, presence of AGM was related to reduced 5-year EFS (73.4±6% *vs* 89.9±3.4%, log-rank, *P*=0.04) and 5-year OS (81.8±5.3% *vs* 96.1±2.2%, log-rank, *P*=0.018) in tumours with higher tumour cell content.

**Figure 1:**
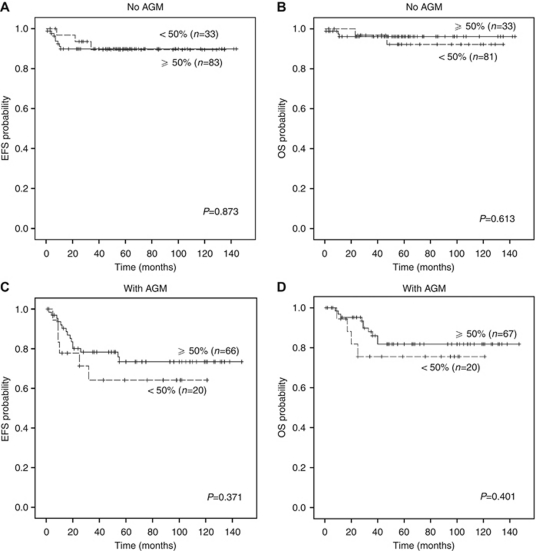
Kaplan–Meier curves showing the effect of tumour cell content on clinical impact of aberrant (numeric or structural) genetic markers (AGM) in neuroblastoma patients. (**A** and **B**) Event-free survival (EFS) and overall survival (OS) of patients with tumours without AGM according to tumour cell content of sample. (**C** and **D**) EFS and OS of patients suffering with tumours with AGM according to tumour cell content of sample.

In order to confirm that there were no differences in patient outcome according to tumour cell content of samples, we then considered AGM and divided the whole cohort into two categories: absence (*n*=139) and presence (*n*=94) of AGM. In tumours without AGM, there were no statistically significant differences in EFS (*P*=0.873) or OS (*P*=0.613) for patients with samples having more or less than 50% tumour cell content (Figures 1A and B). Nor were there statistically significant differences in EFS (*P*=0.371) or OS (*P*=0.401) when considering tumour cell content in tumours with AGM (Figures 1C and D).

## Discussion

Our data clearly demonstrate that there are no differences in prognostic impact of presence of AGM, detected by FISH on TMA including representative tumour areas, according to tumour cell content. In the whole cohort, patients with AGM had significantly worse EFS and OS than patients with no AGM. Furthermore, the poor outcome of patients harbouring tumours with AGM was observed in both tumours subgroups, tumours up to 50% and tumours with >50% of tumour cell content.

In the last two decades, since the initial discovery of the *MYCN* oncogene, many prognostic biomarkers have been proposed for NB. As the genomic type provides additional important prognostic information, the future INRG classification system will rely on the genetic profile of NB tumours rather than on the presence or absence of individual genetic abnormalities (Cohn *et al*, 2009). Whole-genome analysis techniques enable the study of DNA copy number gains and losses over the entire genome in one single experiment and have been used extensively to define the genomic profile of a great variety of solid tumours (Van Roy *et al*, 2009). A novel design of MLPA probes has been developed, which allows MLPA analysis on small amounts of DNA (Sorensen *et al*, 2008). Nevertheless, DNA-based approaches, including MLPA, are not always applicable in clinical practice, because these techniques require DNA obtained from samples with a tumour cell content of >60% (Ambros *et al*, 2009).

Volchenboum *et al* (2009) hypothesised that genome-wide studies of tumour samples may under report genetic changes if the samples are contaminated with normal stromal tissue or heterogeneous tumour elements containing diploid DNA. Recently, our group published a paper comparing relevant genetic aberrations in NB detected by FISH and MLPA in cases with >40% tumour cell content, finding a high concordance between both techniques (Villamon *et al*, 2011). Discrepancies in genetic aberrations detection could be due to intratumoural heterogeneity observed in different tumour areas analysed, as well as the low percentage of neuroblastic cells with the specific DNA copy number alteration or high level of Schwann cells (Villamon *et al*, 2011). Likewise, the SIOPEN (SIOP Europe Neuroblastoma) Biology Committee has published a study examining an inter-technique and inter-laboratory testing of NB MLPA kit. They suggested that a tumour genomic profile with no alterations (flat profile) by MLPA can be caused by tumour cell content below 60%, or by NBs samples with a higher amount of Schwann cells (Ambros *et al*, 2011).

In contrast, FISH technology facilitates single-cell genetic analysis of target regions for investigating cell heterogeneity within tumours. The use of FISH enables the detection of loss or gain of genetic material and reveals rearrangements unsuspected by high-resolution techniques in samples with up to 50% tumour cell content; samples with necrotic, haemorrhagic and/or calcification areas, or samples with a large amount of Schwann cells.

In conclusion, FISH is able to diagnose relevant AGM to determine the prognostic category of NB in case of samples with lower tumour cell content, whereas pangenomic/multilocus data provide a whole-genome profile of samples with higher tumour cell content. Genomic instability analysis of all NB samples to determine patient risk, including cases with low tumour cell content, will be necessary to allow more accurate stratification of patients, and thus reduce aggressive therapy without affecting the outcome for patients.
