# Supplementary material for: How to minimise the effect of tumour cell content in detection of aberrant genetic markers in neuroblastoma
Source: Br J Cancer. 2011 Jun 7;105(1):89–92. doi: 10.1038/bjc.2011.188 (PMC3137406; doi:10.1038/bjc.2011.188)
Supplement: Supplementary Table 1 [file bjc2011188x1.doc]

| **Table 1** Clinical characteristics of patients | | | | | | | | | |  |
| --- | --- | --- | --- | --- | --- | --- | --- | --- | --- | --- |
|  |  | | **Up to 50%** | |  | | | **More than 50%** | | |
| **Characteristics** | |  | **Number** | **%** | |  | **Number** | | **%** | |
| *Sex* | |  |  |  | |  |  | |  | |
| Male | |  | 31 | 50.8 | |  | 99 | | 57.6 | |
| Female | |  | 30 | 49.2 | |  | 73 | | 42.4 | |
|  | |  |  |  | |  |  | |  | |
| *Stage* | |  |  |  | |  |  | |  | |
| 1 | |  | 23 | 37.7 | |  | 72 | | 41.9 | |
| 2 | |  | 8 | 13.1 | |  | 18 | | 10.5 | |
| 3 | |  | 8 | 13.1 | |  | 36 | | 20.9 | |
| 4 | |  | 14 | 23 | |  | 25 | | 14.5 | |
| 4s | |  | 3 | 4.9 | |  | 16 | | 9.3 | |
| Not known | |  | 5 | 8.2 | |  | 5 | | 2.9 | |
|  | |  |  |  | |  |  | |  | |
| *Age at diagnosis* | |  |  |  | |  |  | |  | |
| <18 months | |  | 29 | 47.5 | |  | 128 | | 74.4 | |
| ≥18 months | |  | 31 | 50.8 | |  | 43 | | 25.0 | |
| Not known | |  | 1 | 1.6 | |  | 1 | | 0.6 | |
|  | |  |  |  | |  |  | |  | |
| *Histopathology* | |  |  |  | |  |  | |  | |
| NB | |  |  |  | |  |  | |  | |
| Undiff, poorly diff. | |  | 22 | 36.1 | |  | 151 | | 87.7 | |
| Differentiating | |  | 4 | 6.6 | |  | 11 | | 6.4 | |
| NOS | |  | 12 | 19.7 | |  | 6 | | 3.5 | |
| GNB | |  |  |  | |  |  | |  | |
| intermixed | |  | 9 | 14.8 | |  | 1 | | 0.6 | |
| nodular | |  | 3 | 4.9 | |  | 1 | | 0.6 | |
| Ganglioneuroma | |  | 11 | 18 | |  | 2 | | 1.2 | |
|  |  | |  |  | |  |  | |  | |

Abbreviations: NB = neuroblastoma; undiff = undifferentiated; diff = differentiated; NOS = not otherwise specified; GNB = ganglioneuroblastoma.
